# A cloud-native framework for seismic waveform data quality assessment: Performance evaluation under equivalent resource constraints

**DOI:** 10.1371/journal.pone.0357267

**Published:** 2026-09-03

**Authors:** Yang Li, Danning Wang, Runyuan Dong, Chen Cheng, Xiuping Zhang, Wenqing Wang, Chuanjin Liu

**Affiliations:** The Second Monitoring and Application Center, China Earthquake Administration, Xi’an, China; Xidian University, CHINA

## Abstract

The rapid growth of seismic waveform data requires efficient and elastic processing, but virtual-machine-based architectures often tie workload execution to coarse resource units and rigid scaling policies. To address these limitations, we propose a Kubernetes- and Docker-based cloud-native framework for real-time seismic waveform data quality assessment. The framework organizes the processing logic into three tiers: infrastructure and orchestration, elastic scaling policy, and data quality assessment services. It further introduces an Adaptive Elastic Scheduling Algorithm (AESA) that combines CPU and memory high-watermark triggers with task-lifecycle-aware contraction for containerized computation of key quality indicators, including gap count, gap duration, and percent of availability. Under equivalent hardware resource constraints, the cloud-native implementation reduced mean response latency by 47.27% at 20 requests per second and 47.26% at 50 requests per second compared with the VM-based control group, corresponding to an approximately 1.90-fold speedup in both load regimes. These results indicate that, for the tested gap-count, gap-duration, and availability workloads, cloud-native orchestration can improve latency without changing the quality-metric code. The evaluation is limited to the specified workload, resource budget, and two traffic levels.

## 1 Introduction

High-quality seismic observation data serve as the cornerstone of earthquake disaster mitigation and scientific research. With the global transition toward digitized and high-density seismic networks, exemplified by the implementation of China’s National Seismic Intensity Quick Report and Early Warning Project, the number of observation stations has grown from thousands to tens of thousands, pushing data volumes into the petabyte era [1]. This growth creates two challenges for monitoring systems: earthquake early warning and real-time focal mechanism solutions demand second-level latency in data quality control, while seismic waveform streams exhibit tidal patterns and burstiness, particularly during aftershock sequences following major events.

However, traditional processing architectures, largely dependent on high-performance computing clusters or static Virtual Machines (VMs), are inherently rigid. Such architectures face a critical dilemma: overallocating resources leads to substantial waste during off-peak periods, while resource constraints result in processing delays or backlogs during traffic spikes. Consequently, achieving high-throughput and low-latency computation of quality metrics under limited resource constraints has become a pivotal issue for next-generation seismic monitoring systems.

To address computing and storage bottlenecks, cloud computing technologies offer promising new solutions [2]. While cloud storage and serverless architectures have been explored for large-scale seismic imaging and offline deep learning tasks [3], research focused on “real-time streaming data quality monitoring under massive data volumes” remains insufficient. Existing Quality Control (QC) tools [4] are predominantly restricted to standalone offline modes and lack horizontal scalability. Furthermore, general-purpose cloud-native elasticity strategies often lack mathematical scheduling models tailored to the specific load characteristics of seismic waveform streams. Consequently, it is difficult to balance system responsiveness and resource utilization in resource-constrained scenarios, such as edge computing nodes [5].

Critical challenges in seismic data processing involve containerizing complex waveform metric algorithms and quantifying the actual performance gains of cloud-native architectures compared with traditional VM setups under high-concurrency conditions [6]. Recently, cloud-native technologies, especially Kubernetes-based container orchestration and microservice architectures, have provided new opportunities to resolve the resource-efficiency conflict [7]. However, simply containerizing existing algorithms does not equate to efficient cloud-native computing. The core challenge lies in reducing the performance bottlenecks of traditional virtualization through workflow decomposition and scheduling optimization within strict iso-resource constraints [8]. This requires not only decoupling the logic of seismic quality metrics into microservices but also establishing a model capable of sensing waveform stream load fluctuations and dynamically adjusting computational instances to approach second-level computing-power alignment [9].

This paper presents a cloud-native framework specifically engineered for seismic waveform data quality assessment, incorporating an Adaptive Elastic Scheduling Algorithm (AESA) driven by real-time workload characteristics. Rather than proposing new seismic quality metrics, this research contributes a computing and scheduling model that links bursty seismic waveform streams with container replica lifecycles. By decoupling conventional geometric indicators–such as number of gaps, duration of gaps, and percent of availability–into containerized microservices, the proposed framework restructures the computation workflow to support fine-grained, high-concurrency processing. Furthermore, by integrating real-time telemetry with task completion events, AESA optimizes the distribution and retirement of shard-based processing tasks. This approach uncovers latent computing capacity within existing infrastructure and achieves measurable performance gains without additional hardware investment.

Our contributions are summarized as follows:

- We developed a decoupled, layered cloud-native computing architecture that decomposes waveform quality assessment tasks into stateless microservices. By using Docker containers rather than VM-level deployment, the framework reduces operating-system-level resource redundancy while preserving the original quality-metric logic.
- We formulated an adaptive elastic scheduling model for bursty seismic waveform streams. The model extends the Kubernetes Horizontal Pod Autoscaler (HPA) strategy by combining CPU/memory high-watermark triggers with task-lifecycle-aware contraction, thereby making the scaling thresholds explicit strategy parameters rather than undocumented assumptions.
- We designed an iso-resource comparative experiment under identical hardware constraints. Across six test items and two traffic levels, the cloud-native implementation reduced mean latency by 47.27% at 20 requests per second and 47.26% at 50 requests per second relative to the VM-based control, while using the same quality assessment code and input data.

## 2 Literature review

The modernization of seismic data processing systems involves the integration of advanced computing paradigms, domain-specific applications, and specialized monitoring tools [10]. Currently, research in this field is evolving from static, hardware-dependent infrastructures toward dynamic, software-defined environments. This section reviews the current state of cloud-native elasticity, the adoption of cloud computing in seismology, and the limitations of existing seismic QC systems, highlighting the persistent gaps in handling high-concurrency streaming data under resource-constrained scenarios [11].

### 2.1 Cloud-native elasticity

As cloud computing evolves from Infrastructure as a Service (IaaS) toward the cloud-native paradigm, container orchestration platforms, exemplified by Kubernetes (K8s), have become the de facto standard for distributed systems [12]. The core advantage of cloud-native lies in its fine-grained resource management and automated elastic scaling capabilities [13]. Current research primarily focuses on the HPA mechanism, which adjusts replica counts based on CPU, memory, or custom utilization metrics [8,14,15]. Baliś et al. [16] reported that such elastic strategies can reduce costs for scientific workflows. Other studies on cloud-continuum and serverless orchestration emphasize that scaling policies must be adapted to workload type, latency requirement, and deployment boundary rather than copied directly from web-service scenarios [17,18].

However, the current status of elasticity research is largely tailored to Web services with stable traffic. For seismic data, existing strategies rely on reactive thresholds that suffer from significant “response lag” (Cold-start Latency) during sudden earthquake-induced data bursts. There is a lack of proactive mathematical models that can anticipate the impulsive “tidal effects” of seismic waveform streams, making it difficult to maintain stability during peak traffic [19].

### 2.2 Cloud computing applications in seismology

Seismologists have begun exploring cloud computing to break the bottlenecks of traditional high-performance computing. Ni et al. [20] highlighted how cloud object storage allows researchers to “bring code to the data,” effectively handling PB-scale datasets, while Krauss et al. [21] provided practical guidance for individual seismology researchers moving analysis workflows into cloud environments. Notable implementations include QuakeFlow for automated deep learning deployment [22] and event-driven serverless paradigms for wavefield simulations [3].

Despite these advancements, most current cloud-based seismic applications are optimized for “computation-intensive” offline tasks, such as imaging or model training. Research into “data-intensive” real-time streaming processing remains underdeveloped. Specifically, there is a lack of systematic performance evaluation on how cloud-native architectures can minimize latency for continuous waveform streams when hardware resources are strictly limited.

### 2.3 Seismic data quality monitoring

Data quality control is essential for reliable earthquake early warning. Current systems, such as Pycheron [23], the Pyrocko toolbox [24], and recently reported seismic-accelerometric data quality tools [25], offer robust rule-based algorithms for calculating gaps, availability, and spectral indicators [26]. Deep learning has also been explored for improving seismic data quality analysis [27]. These tools have been widely adopted for research preprocessing and routine network maintenance.

Nevertheless, the major deficiency of existing QC tools is their “rigid” architecture, typically designed for standalone execution or static VM deployment. They lack horizontal scalability, which is critical when processing data from tens of thousands of stations simultaneously. Consequently, when large seismic events trigger a massive surge in data throughput, these systems often face processing backlogs or crashes, failing to meet the “second-level response” demand of modern real-time monitoring.

### 2.4 Research gap and positioning

The literature above indicates that the required algorithmic indicators are relatively mature, whereas the execution architecture for real-time, high-concurrency seismic data quality assessment remains underdeveloped. Existing cloud-seismology studies mainly address storage, imaging, or machine-learning workflows, and existing QC software mainly addresses indicator correctness and offline usability. The unresolved question is how to map bursty station-level waveform workloads onto elastic container replicas under fixed hardware resources. This study is positioned in this gap: it preserves established seismic quality indicators, but redesigns their execution and scheduling layer so that real-time workloads can be processed through a three-tier cloud-native business logic and a workload-aware elastic policy.

## 3 Materials and methods

### 3.1 High-speed cloud-native architecture model

**Fig 1:**
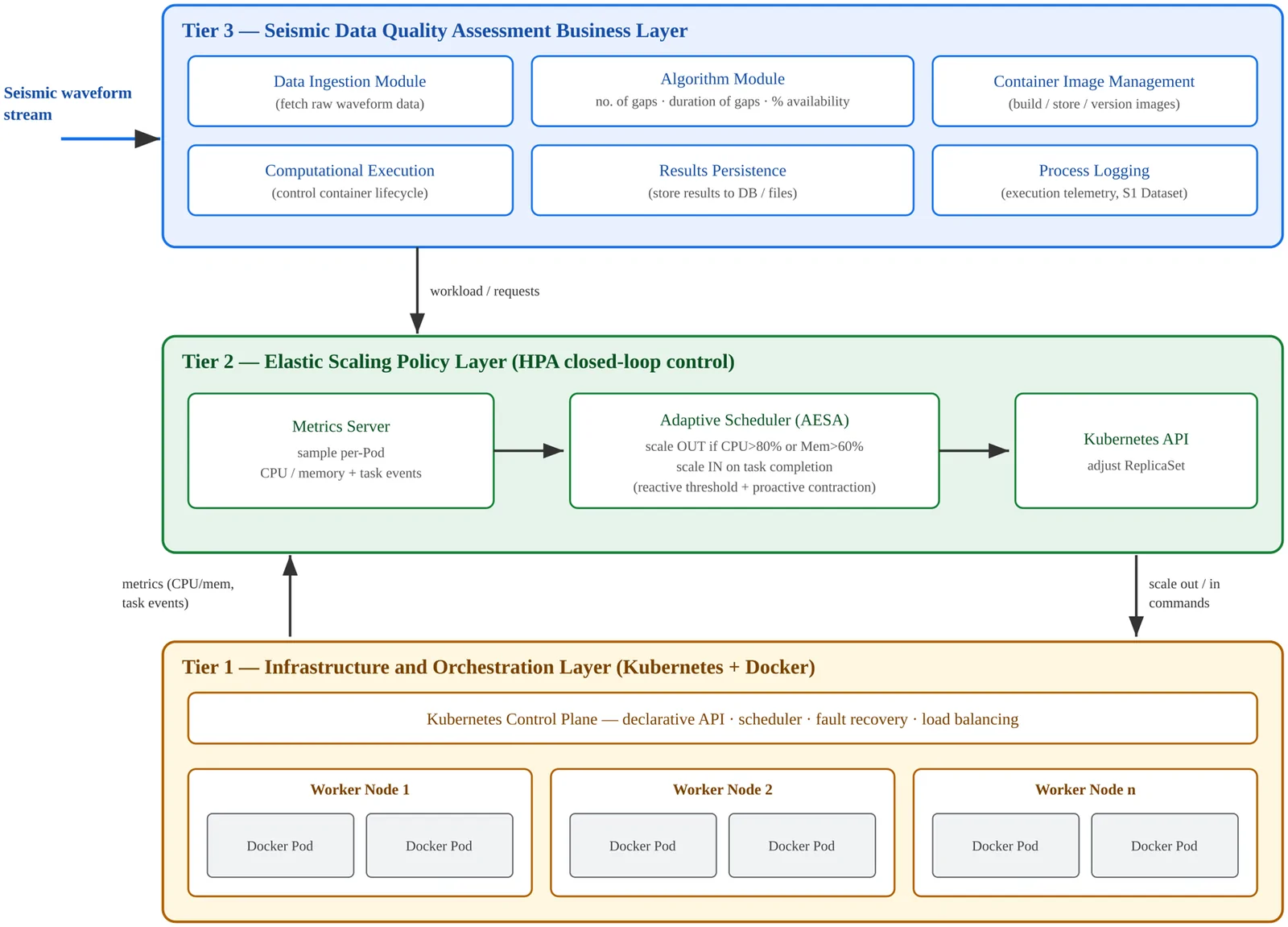
Three-tier business logic of the proposed cloud-native seismic waveform data quality assessment framework. The infrastructure and orchestration layer provides Kubernetes nodes, Docker runtime, service discovery, and metrics collection; the elastic scaling policy layer converts CPU/memory telemetry and task lifecycle events into replica decisions; and the data quality assessment business layer performs data ingestion, containerized indicator calculation, result persistence, and process logging.

To enable real-time, high-throughput evaluation of seismic waveform data quality metrics, we propose a scalable cloud-native architecture based on modular decoupling and layered design principles. The framework is structured into three core layers: the Infrastructure and Orchestration Layer, the Elastic Scaling Policy Layer, and the Business Layer for Data Quality Assessment. Fig 1 explicitly separates these three layers and shows the control loop between resource telemetry, scaling decisions, and data quality services. This multi-layered design supports adaptive resource allocation and containerized processing of continuous seismic data streams.

**Infrastructure and Orchestration Layer:** Serving as the foundational resource base for the system, this layer utilizes Kubernetes as the core orchestration engine to build a high-availability cluster. Through Declarative APIs, the K8s control plane maintains the desired state of the cluster in real-time and manages fault recovery and load balancing across computing nodes. This helps maintain a stable operational environment for upper-layer metric computations. Docker containers serve as the carriers for algorithm execution; core algorithms, such as data percent of availability and number of gaps calculations, are encapsulated into independent microservice units. These units share the host kernel, forming the minimal execution entities of the model.

**Elastic Scaling Policy Layer:** This layer implements a closed-loop feedback control system by integrating the HPA component. It uses the Metrics Server to collect real-time CPU and memory utilization data from computing Pods. The CPU and memory thresholds are not independent assumptions; they are defined as explicit elastic-scaling strategy parameters. Scaling is triggered when CPU utilization exceeds 80% or memory utilization exceeds 60%. The CPU threshold acts as the primary compute-saturation indicator for waveform metric loops, while the lower memory threshold provides an earlier safeguard against page-cache pressure, garbage collection overhead, and container eviction risk in the 1-core/2 GB pod configuration used in the experiment. Conversely, once the metric algorithms complete their execution, the system triggers the termination of redundant Pods and recovers resources. The scheduling algorithm calculates the required number of replicas and starts additional containers to distribute the workload. This mechanism is intended to reduce latency during high-load periods and release idle resources after task completion.

**Seismic Data Quality Assessment Business Layer:** The core functionality is implemented through six specialized modules. The Algorithm Module contains specialized code for seismic quality metrics optimized for containerized deployment, while the Data Ingestion Module acts as an independent unit designed to fetch raw waveform data from various sources including file systems and data services. Supporting these is the Container Image Management Module, which manages the build, storage, and versioning of Docker images containing the algorithm and data logic. The Computational Execution Module orchestrates the lifecycle of computing tasks by controlling container execution and termination, complemented by the Results Persistence Module that handles the systematic storage of computational results into databases or specified file formats. Finally, the Process Logging Module records detailed execution telemetry for subsequent performance analysis, research, and comparative testing.

**Fig 2:**
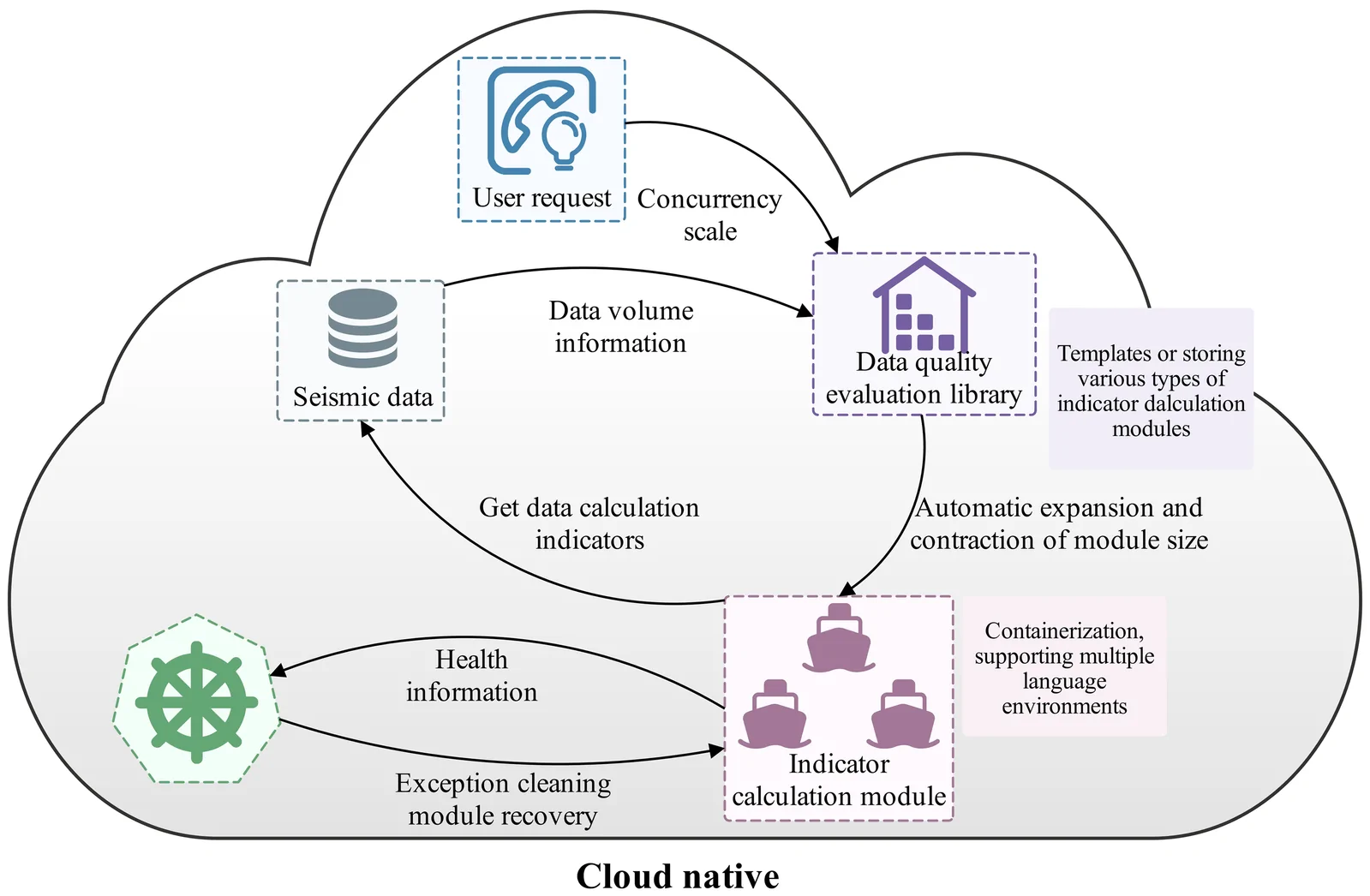
Cloud-native architecture for seismic data quality evaluation. User requests, seismic waveform data, indicator templates, containerized computation modules, and health monitoring form a closed-loop service workflow.

The proposed cloud-native architecture supports scalable seismic data quality evaluation through a modular, containerized design. As illustrated in Fig 2, upon receiving a user request, the system scales according to concurrency levels and retrieves relevant seismic data along with volume information. The data quality evaluation library orchestrates the computation process by selecting indicator calculation modules from a reusable template repository. These modules are deployed in a containerized environment supporting multiple programming languages. The system adjusts the number of running instances based on workload demand, and health monitoring records module status for exception handling and recovery. This closed-loop architecture integrates data ingestion, scaling, and monitoring within a unified cloud-native framework.

**Fig 3:**
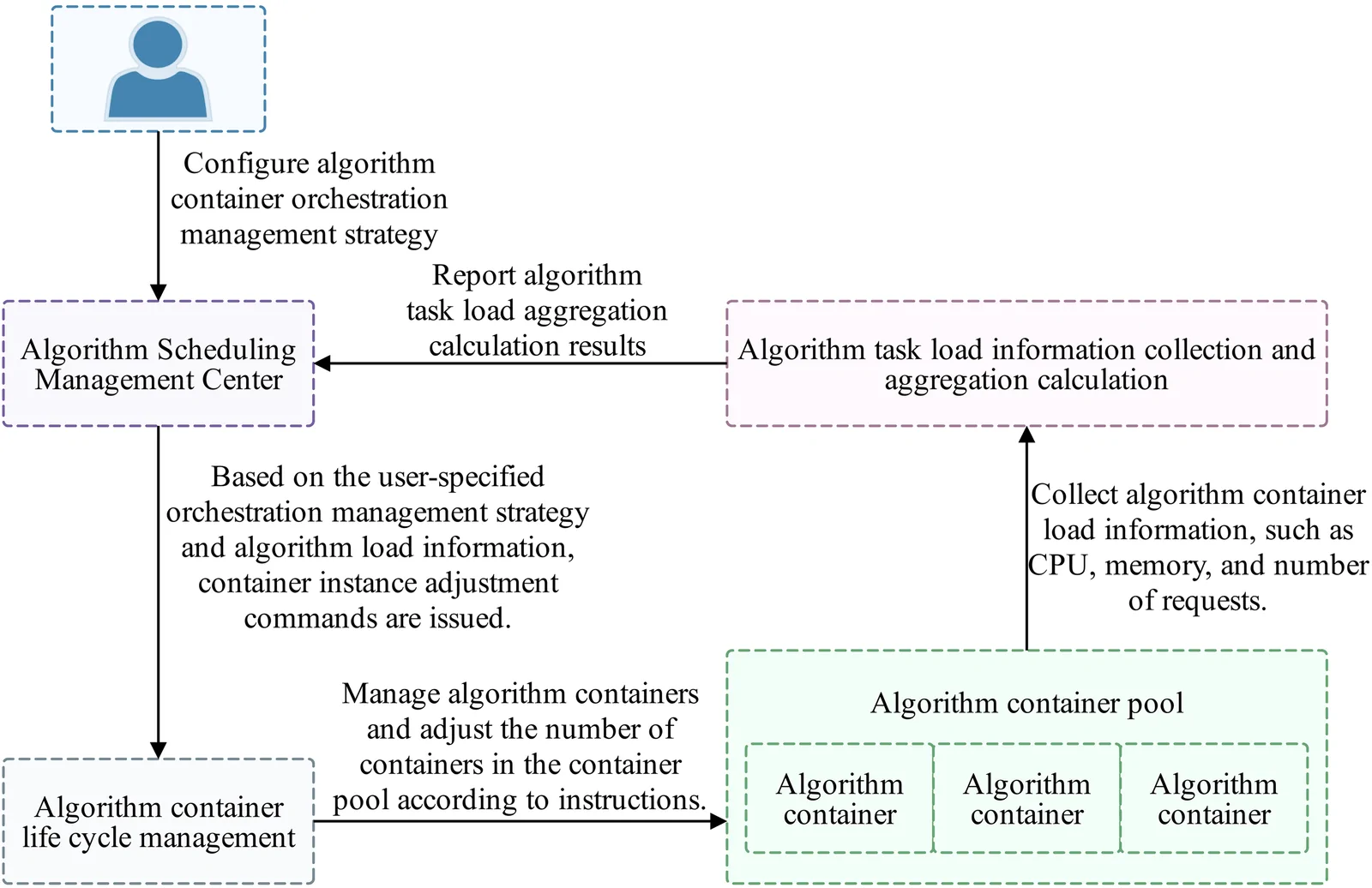
Workflow of the algorithm container orchestration system. The scheduling management center aggregates load metrics, applies the configured scaling strategy, and invokes lifecycle management operations to expand or contract the algorithm container pool.

The algorithm container orchestration mechanism is designed to support dynamic resource allocation and task scheduling in a cloud-native environment. As shown in Fig 3, the process begins with the user configuring the orchestration management strategy, which is then sent to the Algorithm Scheduling Management Center. This center collects real-time load information from the Algorithm Container Pool, such as CPU usage, memory consumption, and request volume, via the Algorithm Task Load Information Collection and Aggregation Calculation module. Based on the user-defined strategy and aggregated load metrics, the center issues container instance adjustment commands. These instructions are executed by the Algorithm Container Lifecycle Management component, which scales the number of running algorithm containers within the pool.

### 3.2 Seismic geometric indices

Seismic waveform geometric indices primarily encompass the number of gaps, the duration of gaps, the number of overlaps, the percent of availability, and the number of samples. This research focuses on the implementation and containerized optimization of three critical indices: the number of gaps, the duration of gaps, and the percent of availability.

**The Number of Gaps (**$N_{gap}$**):** Seismic waveform data are discrete time-series sampled at a constant interval. A “gap” occurs when the time difference between consecutive sampling points deviates from the reciprocal of the sampling rate due to data loss. The number of gaps represents the total number of such interruptions within a specific observation window.

First, we define the Gap Indicator Function Φ(i) for the *i*-th sampling interval:


$$\begin{array}{c} \Phi (i)=\left\{ \begin{array}{cc} 1, & \text{if }|(t_{i+1}-t_{i})-\Delta \tau |>\epsilon \\ 0, & \text{otherwise} \end{array}\right. \end{array}$$ (1)

where $t_{i}$ is the timestamp of the *i*-th sample, Δτ is the theoretical sampling interval, and ϵ is a predefined error threshold. The total gap count $N_{gap}$ is the summation of Φ(i) over the observation period.

**The Duration of Gaps** ($D_{gap}$): the duration of gaps refers to the time elapsed between the timestamp of the first sample following an interruption and that of the last sample prior to the interruption. It quantifies the total period of missing data within the observation window.

The effective missing duration for a single gap event is defined as:


$$\begin{array}{c} d_{i}=(t_{i+1}-t_{i})-\Delta \tau \end{array}$$ (2)

Consequently, sum of the duration of gaps ($D_{gap}$) within the observation window is calculated as:


$$\begin{array}{c} D_{gap}=\sum_{i=1}^{M-1}(\Phi (i)\times d_{i}) \end{array}$$ (3)

where *M* is the total number of expected samples.

**The Percent of Availability** ($P_{availability}$): The percent of availability, ranging from 0% to 100%, represents the coverage of observed data relative to the total expected duration. While a continuous stream yields 100% completeness, any occurrence of gaps reduces this ratio proportionally to the total gap duration.

Let $t_{start}$ and $t_{end}$ be the predefined start and end times of the observation task, respectively. The total planned observation duration is $T_{obs}=t_{end}-t_{start}$. The Percent of Availability ($P_{availability}$) is defined as the percentage of effective observation time relative to the total task duration:


$$\begin{array}{c} P_{availability}=\left(1-\frac{D_{gap}}{T_{obs}}\right)\times 100\% \end{array}$$ (4)

### 3.3 Adaptive elastic scaling strategy

To improve resource matching during workload fluctuation, an automated pod-level scaling mechanism was developed for seismic data quality assessment tasks. By continuously monitoring the average CPU and memory loads of Pod groups executing computational tasks, the system triggers horizontal scaling when predefined thresholds are breached. The parallel deployment across computing nodes is designed to increase metric calculation throughput under load. If total cluster resources approach saturation, the framework can also support node-level expansion, although the present experiments focus on pod-level scaling under a fixed hardware budget.

This scaling mechanism is built upon the Load-Aware HPA concept, integrating the Kubernetes native HPA component to form a closed-loop feedback control system. The Metrics Server performs real-time collection of core resource metrics (e.g., CPU and memory utilization) to establish a quantitative workload assessment model [15]. In the Kubernetes HPA design, multiple resource metrics can jointly drive replica adjustment, and the highest recommended replica count is selected when several metrics are configured [14]. Following this multi-metric logic, we define CPU utilization >80% or memory utilization >60% as the scale-out rule. These two thresholds are therefore part of the elastic scaling strategy: the CPU high-watermark captures compute saturation in iterative waveform quality calculations, while the lower memory threshold reserves headroom for buffered waveform segments, temporary arrays, and runtime overhead in each 2 GB container. Upon triggering, the scheduling algorithm calculates the required number of additional Pod replicas based on current workload levels and pre-set policies, then deploys new instances to distribute the load. Task completion serves as the contraction trigger for decommissioning idle containers. This design aligns resource supply with service demand at the pod level; the measured latency effects of this design are reported in the Results section.

Given the pronounced “tidal patterns” and burstiness of seismic waveform streams, traditional static allocation or single-metric (CPU-only) scaling may fail to balance resource utilization with real-time requirements. To address this, we propose an Adaptive Elastic Scheduling Algorithm. This algorithm first provides a mathematical formalization of seismic stream workloads and subsequently constructs a hybrid scheduling model that integrates “reactive expansion” with task-lifecycle-aware contraction. Under iso-resource constraints, this model is designed to improve computational throughput and system responsiveness.

Seismic monitoring data streams exhibit non-linear characteristics, where baseline loads are overlaid with sudden bursts. We formally define the total computational load pressure on the system at time *t*, $L_{total}(t)$, as:


$$\begin{array}{c} L_{total}(t)=\beta \cdot L_{base}(t)+\alpha \cdot \sum_{i=1}^{k}E_{i}(t-\tau_{i})+\varepsilon (t) \end{array}$$ (5)

where $L_{base}(t)$ denotes the baseline load generated by routine continuous waveform processing, β is the weighting coefficient of the baseline component, $E_{i}(t-\tau_{i})$ represents the burst load contributed by the *i*-th seismic event with onset delay $\tau_{i}$, *k* is the number of concurrent burst events within the observation window, α is the amplification coefficient of the event-driven component, and ε(t) is a stochastic disturbance term capturing measurement and scheduling jitter.

Traditional Kubernetes HPA strategies typically make decisions based on smoothed utilization averages. While this averaging suppresses the interference of ε(t), it can lead to response lag for sudden bursts *E*(*t*) and may keep idle resources occupied after an event because of stabiliza*t*ion and cooldown behavior.

Let $\theta_{cpu}=0.80$ and $\theta_{mem}=0.60$ denote the CPU and memory high-watermark thresholds, respectively. The normalized trigger intensity is defined as:


$$\begin{array}{c} U_{trigger}(t)=\max\left(\frac{U_{cpu}(t)}{\theta_{cpu}},\frac{U_{mem}(t)}{\theta_{mem}}\right) \end{array}$$ (6)

Let $P_{t}=\{p_{1},p_{2},...,p_{n}\}$ be the set of active computing replicas at time *t*, with the current *t*otal number of replicas denoted as $N(t)=|P_{t}|$. The target number of replicas, $N_{target}(t+\Delta t)$, is determined by the following piecewise control function:


$$\begin{array}{c} N_{target}(t+\Delta t)=\left\{ \begin{array}{cc} N(t)+\lceil \kappa \cdot (U_{trigger}(t)-1)\cdot N(t)\rceil & \text{if }U_{trigger}(t)>1\\ N(t)-\sum_{j=1}^{m}\delta (Task_{j}) & \text{if }\exists Task_{j}\in \Phi_{completed}\\ N(t) & \text{otherwise} \end{array}\right. \end{array}$$ (7)

**Algorithm logic and feedback control:** The first part of the equation describes the strategy for load surges. When real-time CPU utilization exceeds 80% or memory utilization exceeds 60%, $U_{trigger}(t)>1$ and the algorithm triggers expansion. By introducing an aggressive coefficient κ≥1, the expansion step is non-linearly amplified so that computing supply can respond to the rising edge of *E*(*t*) and reduce backlog risk. Unlike traditional passive scaling that waits for the average load to drop, *t*his model introduces a Task Lifecycle Binding Mechanism. Here, $\Phi_{completed}$ represents the set of completed tasks within the current window, and $\delta (Task_{j})$ is the task completion indicator function:


$$\begin{array}{c} \delta (Task_{j})=\left\{ \begin{array}{cc} 1, & \text{Completed}\\ 0, & \text{In progress} \end{array}\right. \end{array}$$ (8)

Once a station’s data processing task is finished, the algorithm sends a termination signal directly to the corresponding computing container $p_{j}$ via the Kubernetes API, immediately releasing CPU and memory resources back to the pool.

**Dual-channel closed-loop feedback control:** The system operates on a “Perception-Decision-Execution” control loop. **State Sensing Layer:** It maintains two input streams: one collecting real-time cluster CPU/memory metrics via Metrics Server to quantify computing load, and another monitoring the Kubernetes API Server event stream to capture the lifecycle status (e.g., Pending or Succeeded) of seismic waveform tasks.

**Decision Core:** The scheduler performs shunting decisions. Upon detecting a CPU or memory load breach, it activates the up-scaling path, calculates the normalized trigger intensity, and generates expansion commands that suppress sudden traffic bursts. Upon receiving a “task completion” signal, it immediately activates the down-scaling path, triggering termination without waiting for the HPA cooldown window.

**Execution Layer:** Issues commands to adjust the ReplicaSet scale, linking the resource supply curve to the seismic load curve and supporting high-concurrency throughput within the configured resource budget.

### 3.4 Experimental design and performance evaluation framework

**Fig 4:**
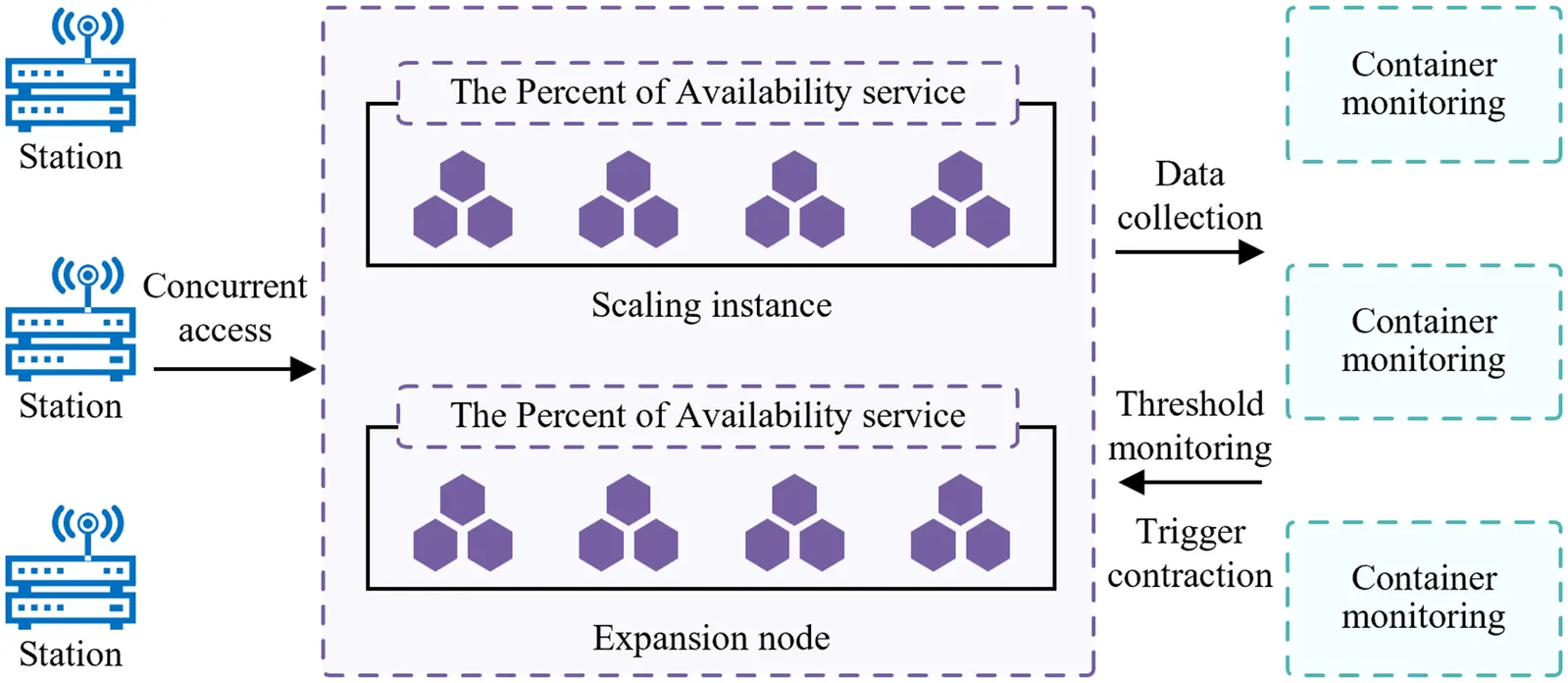
Dynamic scaling architecture for the completeness-rate service. The architecture links incoming concurrency, Metrics Server telemetry, HPA-based scale-out, task-completion-triggered scale-in, and persistent result output.

To quantify the effectiveness of the cloud-native architecture in seismic data processing scenarios, this study establishes a standardized Empirical Evaluation Framework, as illustrated in Fig 4. Based on the principle of “iso-resource” control, the framework uses real-world data quality assessment workloads of varying intensities to compare the cloud-native container architecture with the traditional VM architecture in terms of response latency, speedup, and latency distribution.

(1) Dataset selection The experimental dataset consists of a baseline sample of 1,000 seismic stations randomly selected from the 1,107 stations in the China Seismic Network. The data spans a full 24-hour period (UTC) on July 14, 2025. This dataset includes stable background noise, random gaps caused by network jitter, and fragments of missing data of varying lengths. Such mixed characteristics provide a practical workload for testing boundary handling when processing “dirty data.”
(2) Experimental setup To eliminate interference from hardware performance variations, a strict iso-resource control strategy was implemented under an identical hardware environment by dividing the testing into two physically isolated groups consisting of a Control Group (CG) representing the traditional monolithic VM deployment mode and an Experimental Group (EG) representing the cloud-native architecture. Reflecting the original design goal of execution without modifying existing business code, the cloud-native group utilizes two container instances with each allocated exactly half of the hardware resources of a single VM to execute a testing suite comprising six items derived from two iterations of the gap count, gap duration, and completeness calculation algorithms.

**Fig 5:**
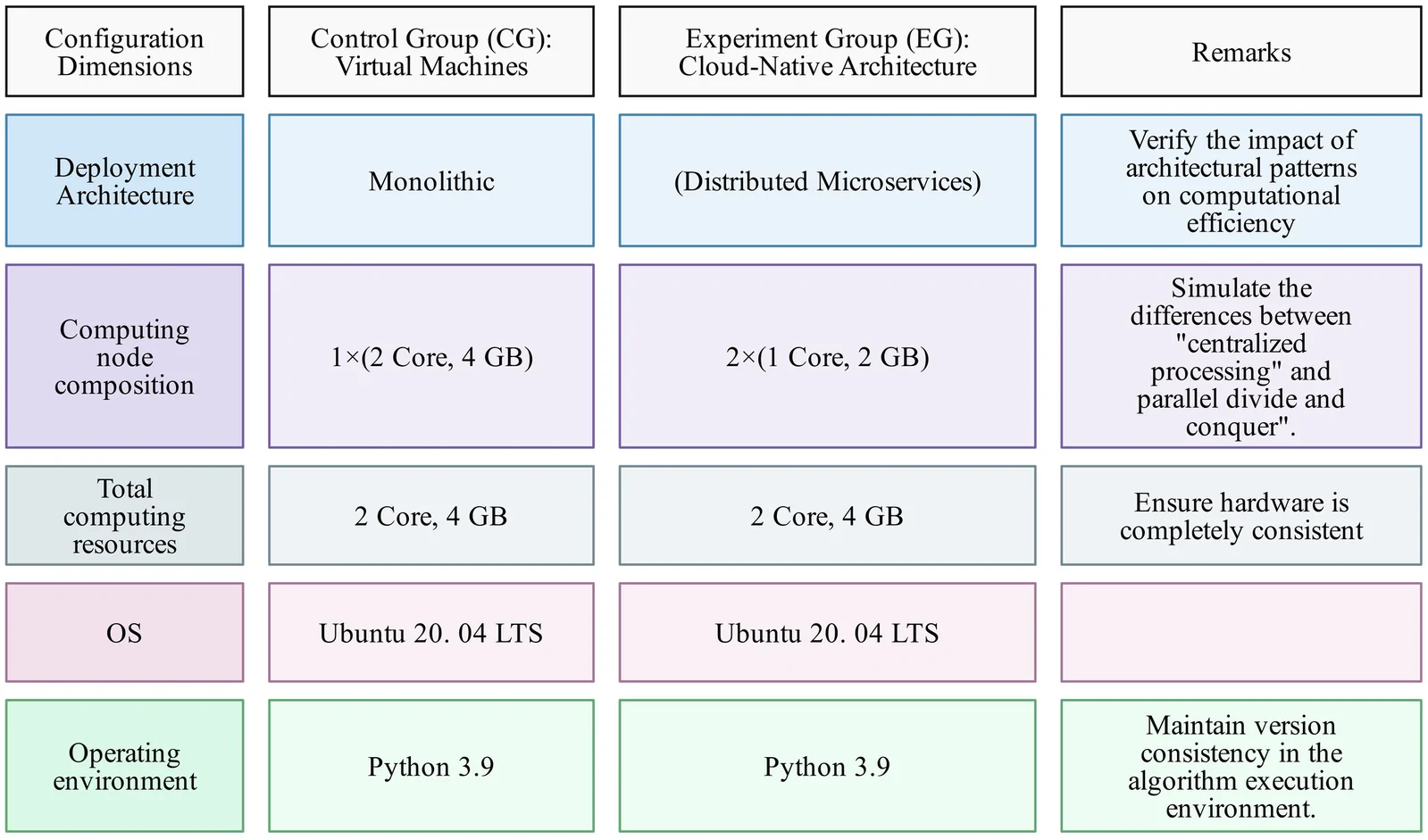
Comparison of experimental configurations between the Control Group and the Experiment Group. The CG uses a single 2-core/4 GB VM-style instance, whereas the EG uses two 1-core/2 GB containerized instances under the same total resource budget.

The experiment compares task completion times inside and outside the proposed system framework within a uniform configuration of 2-core CPU and 4 GB RAM by deploying two algorithm instances each allocated 1-core CPU and 2 GB RAM for the EG and a single instance allocated the full 2-core CPU and 4 GB RAM for the CG. The same quality assessment code and input data were used in both groups; only the deployment and scheduling architecture differed. In the EG, the HPA policy used the CPU > 80% or memory >60% rule described above, making the thresholds part of the experimental elastic scaling strategy. Both groups were subjected to workloads of 20 requests per second and 50 requests per second covering a total of 1,000 stations and 3,000 computational requests to simulate computational efficiency under low-traffic and high-traffic conditions as detailed in the topological configurations summarized in Fig 5.

(3) Implementation and measurement The experimental environment ran on CentOS 7 hosts, with Docker 20.10.11 as the container runtime and Kubernetes v1.21.0 providing orchestration, including the Metrics Server and the HPA components; the CG virtual-machine image and the EG container images were both based on Ubuntu 20.04 LTS and executed the algorithms with Python 3.9, as summarized in Fig 5. Each of the three geometric quality indicator algorithms was packaged as an independent container image, and the six test items correspond to two evaluation rounds of the three indicator services. A load generator issued requests at 20 and 50 requests per second against the 1,000 selected stations, for a total of 3,000 computational requests per configuration. Per-request processing times were recorded by the process logging module described in the business layer; these records are the source of the summary statistics reported in the Results section and provided in S1 Data.

## 4 Results

**Fig 6:**
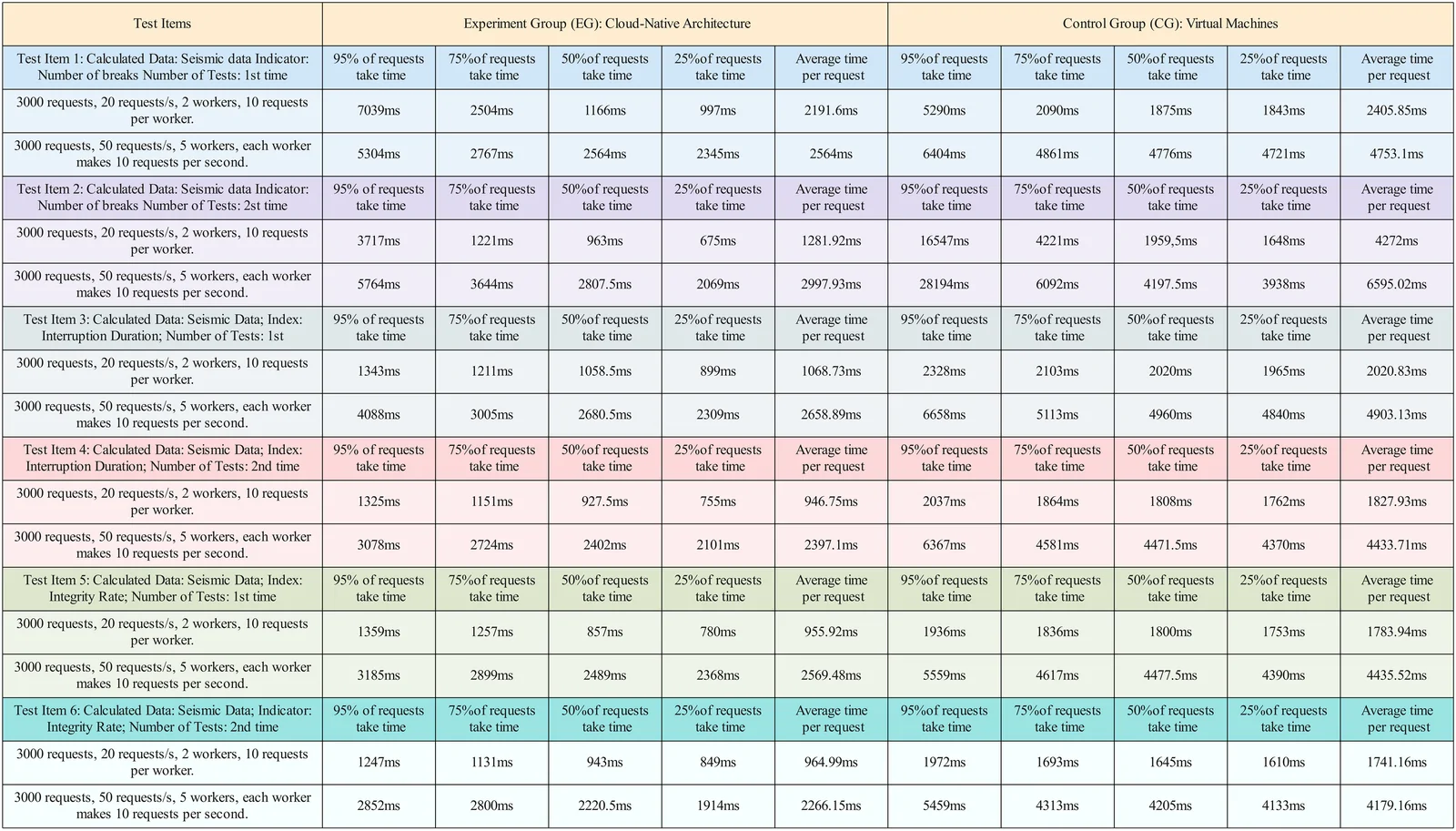
Experimental results comparing the cloud-native Experiment Group (EG) with the monolithic Control Group (CG) across six test scenarios. The table reports 95th, 75th, 50th, and 25th percentile response times and mean response time for each workload and group.

To evaluate the computational performance of the proposed cloud-native framework for real-time seismic waveform data quality assessment, this study conducted comparative tests benchmarking the EG against the CG. Evaluation metrics included average response latency, speedup ratio, and latency distribution across six test items subjected to concurrency levels of 20 and 50 requests per second. The test results are shown in Fig 6.

### 4.1 Overall computational efficiency assessment

**Fig 7:**
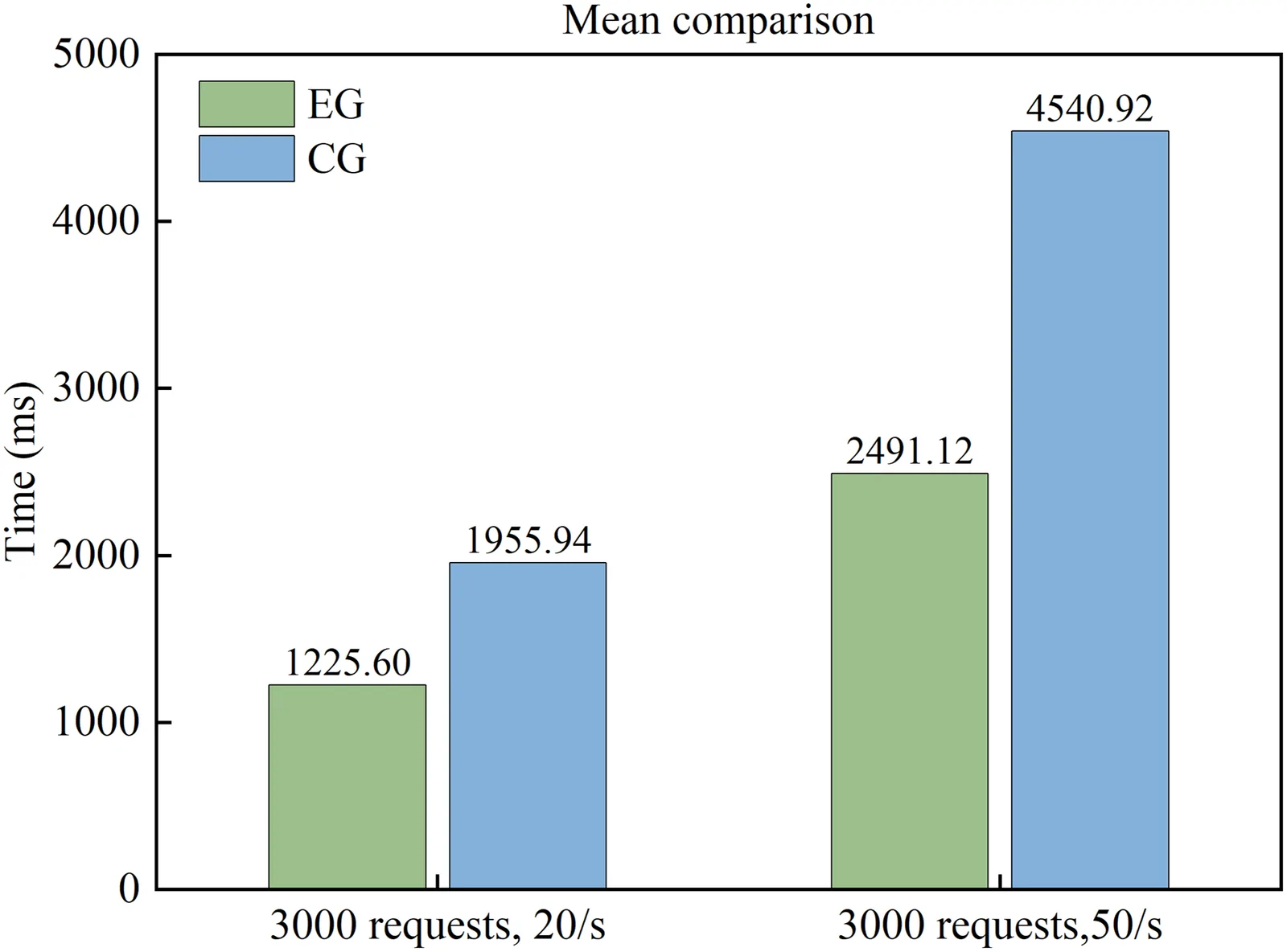
Mean processing time comparison between the EG and CG. Each group handled 3000 total requests under 20 and 50 requests per second. Values were calculated from the six test-item means reported in S1 Data.

The baseline performance evaluation focused on the average processing time for gap counts, gap durations, and completeness rates under the two traffic settings. As shown in Fig 7, the Experiment Group (EG), based on a cloud-native microservices architecture, had lower mean latency than the Control Group (CG) using a monolithic VM deployment. Based on the reconciled values provided in S1 Data, at 20 requests per second the EG achieved a mean response time of 1234.98 ms, compared with 2341.95 ms in the CG, corresponding to a 47.27% reduction in mean latency and a 1.90-fold speedup. At 50 requests per second, the EG achieved a mean response time of 2575.59 ms, while the CG required 4883.27 ms, corresponding to a 47.26% latency reduction and a 1.90-fold speedup. These results show lower mean response latency for the cloud-native deployment under identical hardware constraints.

Three observations are drawn from the six test items. First, lower mean latency was achieved through containerized replica expansion and automated scaling without modifying the core calculation code. Second, the approximately 1.90-fold speedup was slightly below the ideal two-replica ratio, indicating that container orchestration and scheduling overhead remain measurable. Third, the latency distributions suggest that distributed execution can reduce blocking in several high-load cases, but direct queue-level instrumentation would be needed to quantify this mechanism.

### 4.2 Analysis based on traffic concurrency

**Fig 8:**
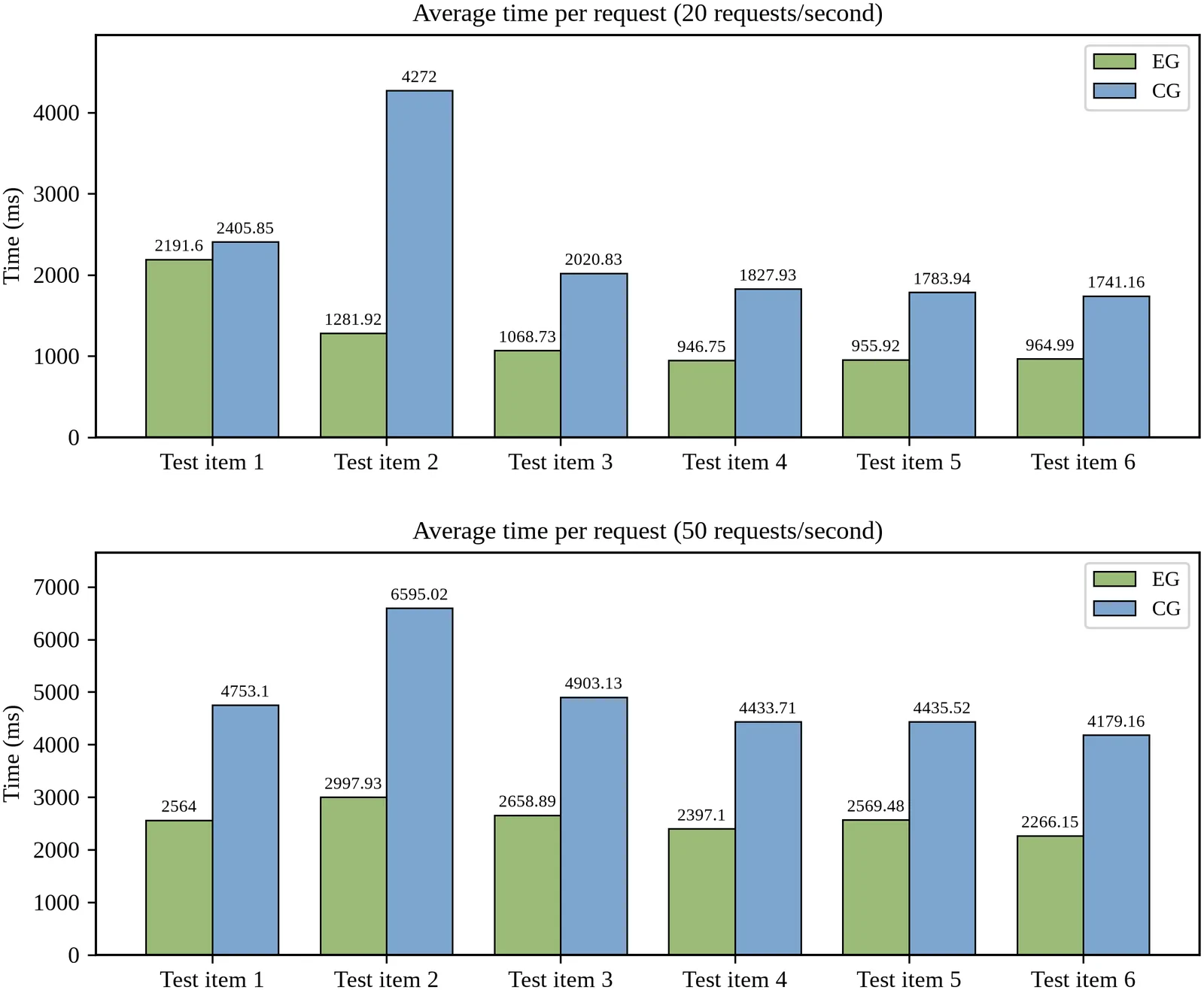
Average time per request for six test items. Results are shown under two concurrency levels: 20 and 50 requests per second.

A comparative analysis under varying traffic loads shows that the cloud-native architecture had lower average processing times across the tested workloads. As illustrated in Fig 8, at 20 requests per second the EG achieved lower average processing times than the CG across all six test items. Test Items 1 and 2 showed larger tail-latency fluctuation than the other items, which is discussed below, but their mean values still favored the EG. At 50 requests per second, the EG maintained its latency advantage in every test item.

Further analysis indicates that while the EG exhibited higher latency fluctuation at 20 requests per second in some test items, the CG showed greater variability at 50 requests per second. This pattern suggests that the VM-based deployment was more sensitive to stress in the tested high-load condition. The consistent mean-latency reductions across multiple test items support the use of distributed microservice deployment for these seismic quality assessment workloads.

### 4.3 Detailed test item analysis

**Fig 9:**
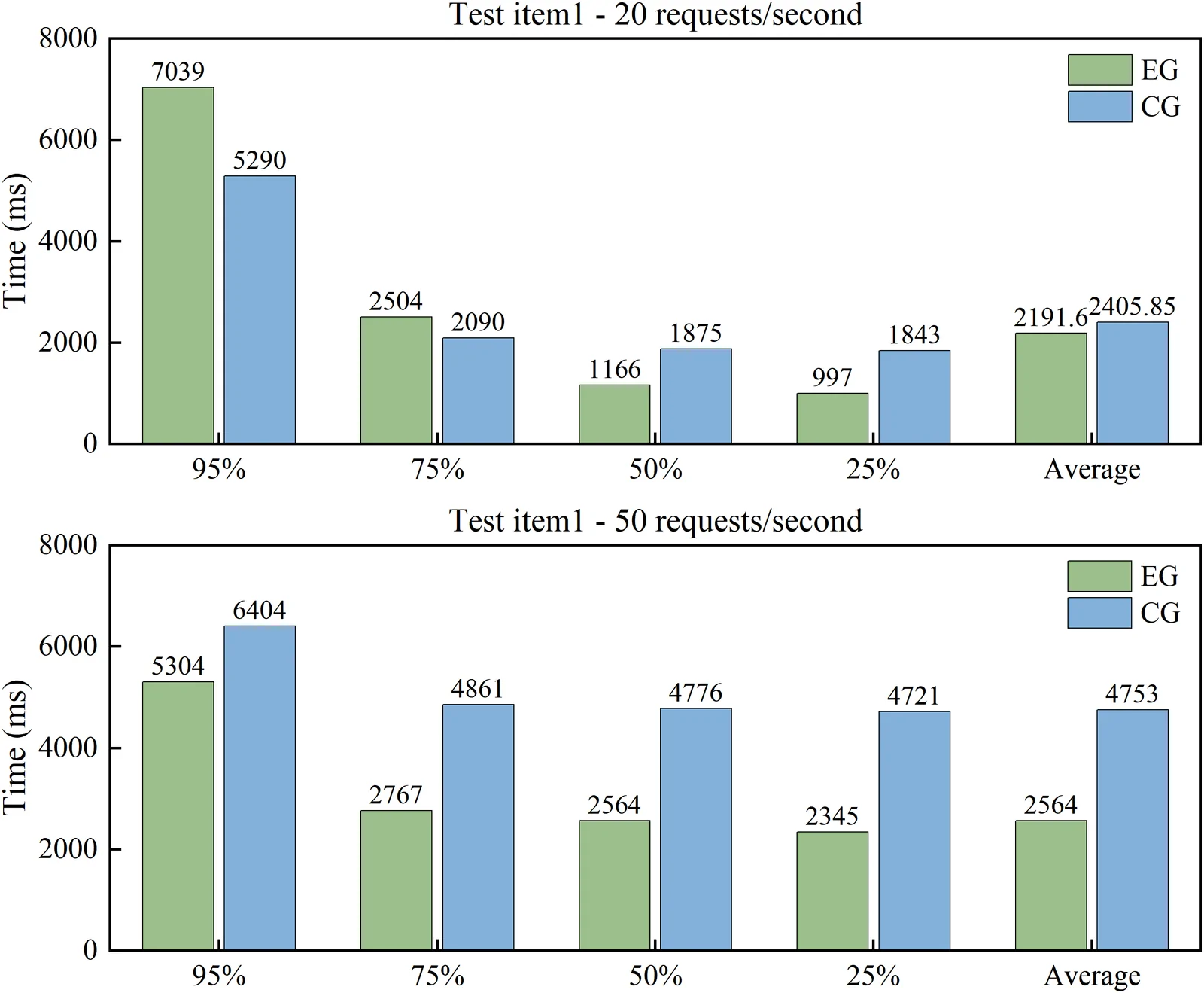
Latency distribution of Test Item 1. Response times are compared between EG and CG under traffic loads of 20 and 50 requests per second.

Fig 9 shows the performance of Test Item 1 under the two traffic loads. At 20 requests per second, the EG had a lower mean latency than the CG (2191.60 ms vs. 2405.85 ms), but its tail latency was higher at the 75th and 95th percentiles. At 50 requests per second, the EG had lower latency at every reported percentile and a lower mean latency than the CG (2564.00 ms vs. 4753.10 ms). This test item therefore supports the mean-latency advantage of the cloud-native deployment, while also showing that tail-latency behavior can vary by workload and load level.

Comparing the 20 and 50 requests per second scenarios further shows that higher traffic increased baseline per-task latency in both architectures. The distributional difference between the two scenarios indicates that mean latency alone is insufficient for evaluating elastic scheduling; percentile-level behavior is also needed.

**Fig 10:**
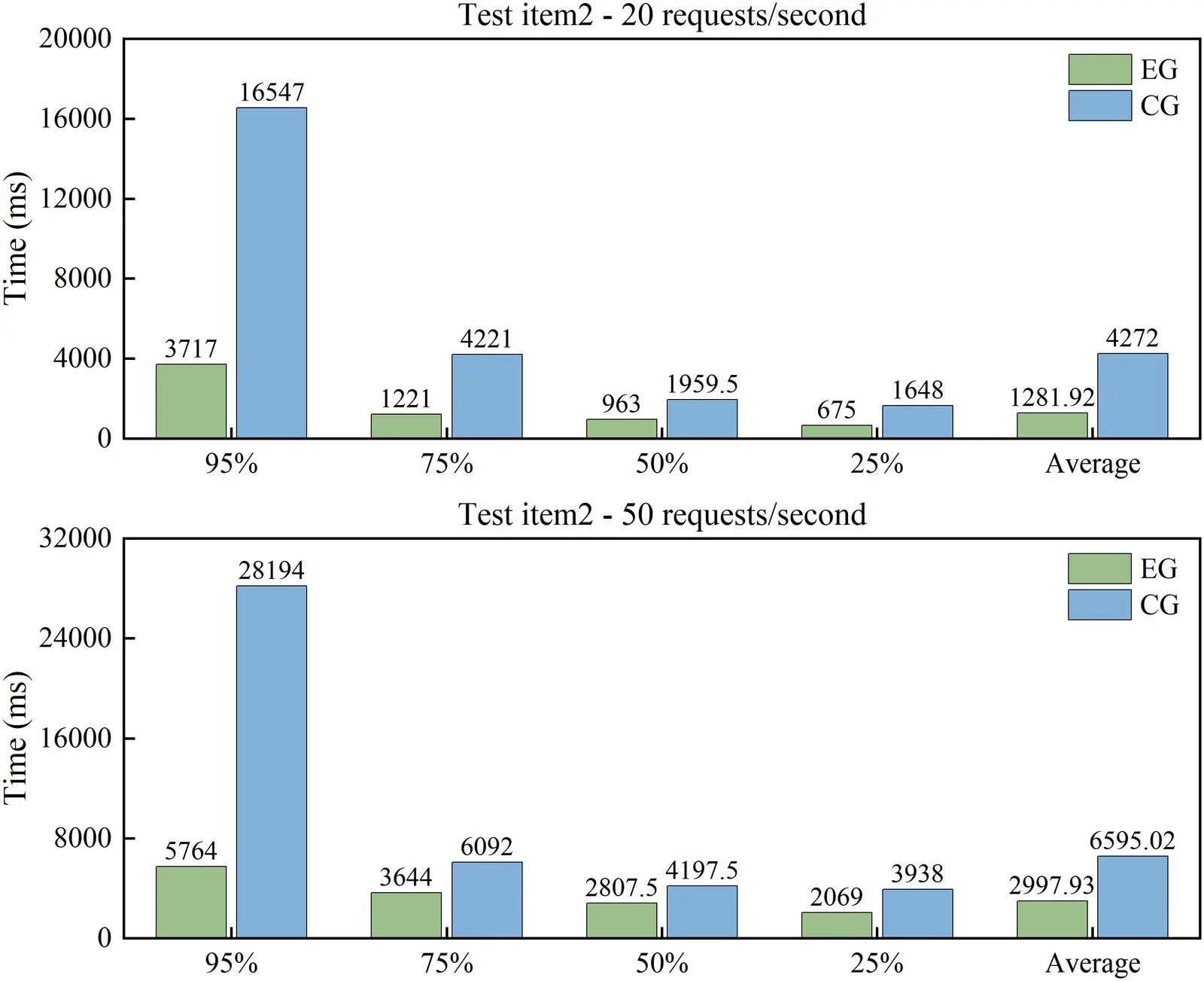
Latency distribution of Test Item 2. Response times are compared between EG and CG under traffic loads of 20 and 50 requests per second.

The performance characteristics of Test Item 2 are shown in Fig 10. At 20 requests per second, the EG mean latency was 1281.92 ms, whereas the CG mean latency was 4272.00 ms. The CG also showed a sharp 95th-percentile increase to 16547 ms. At 50 requests per second, the EG again had lower mean latency (2997.93 ms vs. 6595.02 ms), while the CG 95th percentile increased to 28194 ms. These values indicate a pronounced tail-latency problem in the VM-based deployment for this test item.

Because the VM tail values disproportionately affect the mean, the advantage in Test Item 2 should be interpreted together with the percentile distribution. The result supports lower EG latency, but it also identifies tail latency as the main source of performance divergence.

**Fig 11:**
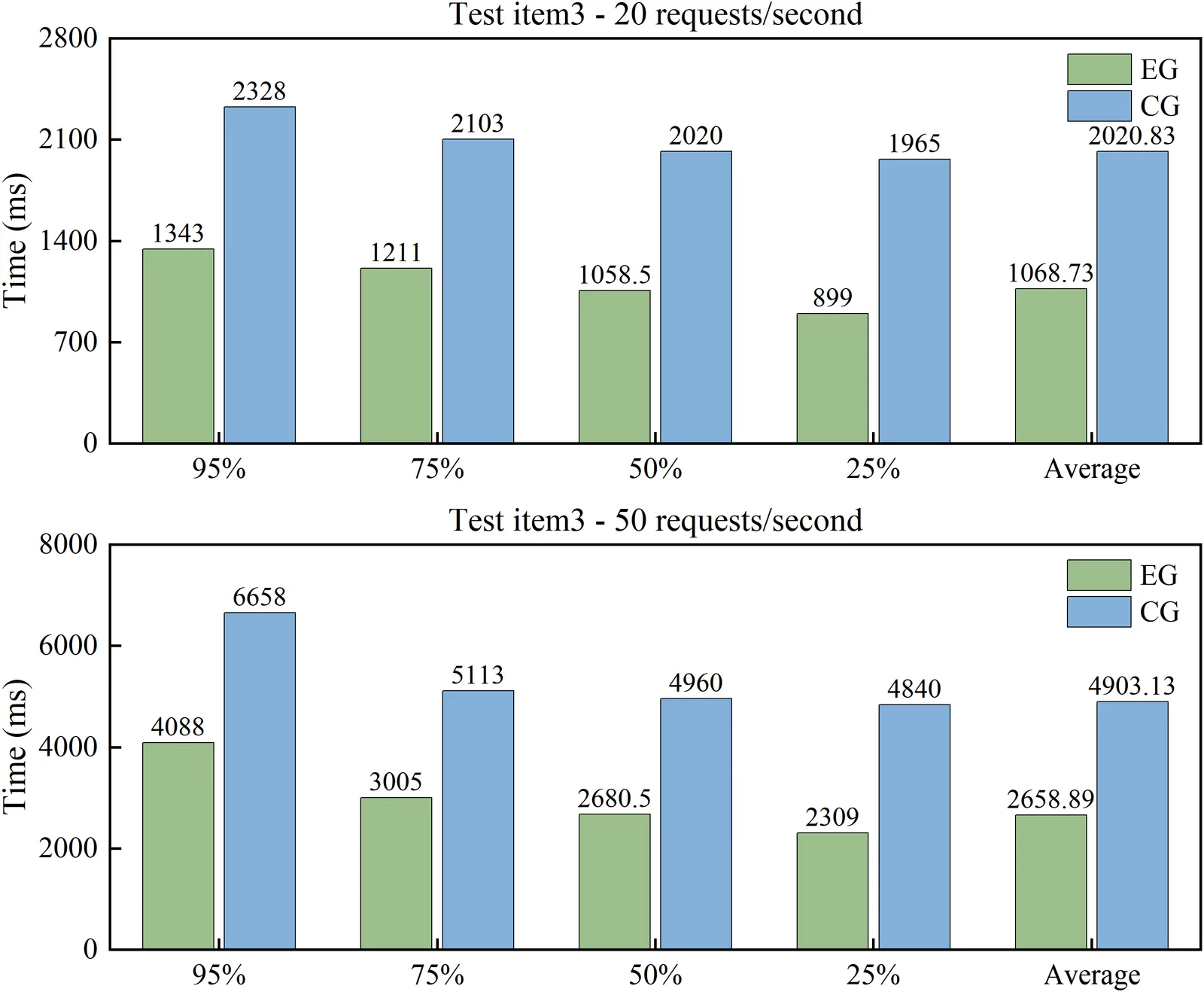
Latency distribution of Test Item 3. Response times are compared between EG and CG under traffic loads of 20 and 50 requests per second.

The performance of Test Item 3 was evaluated under both traffic loads. As shown in Fig 11, at 20 requests per second the EG mean latency was 1068.73 ms, compared with 2020.83 ms in the CG. At 50 requests per second, the EG mean latency was 2658.89 ms, compared with 4903.13 ms in the CG. Both architectures showed smoother percentile distributions than those observed in Test Items 1 and 2.

The increase from 20 to 50 requests per second was reflected in both architectures, but the EG retained lower latency under the same input data and total hardware budget. This result supports the overall mean-latency trend without relying on an isolated outlier.

**Fig 12:**
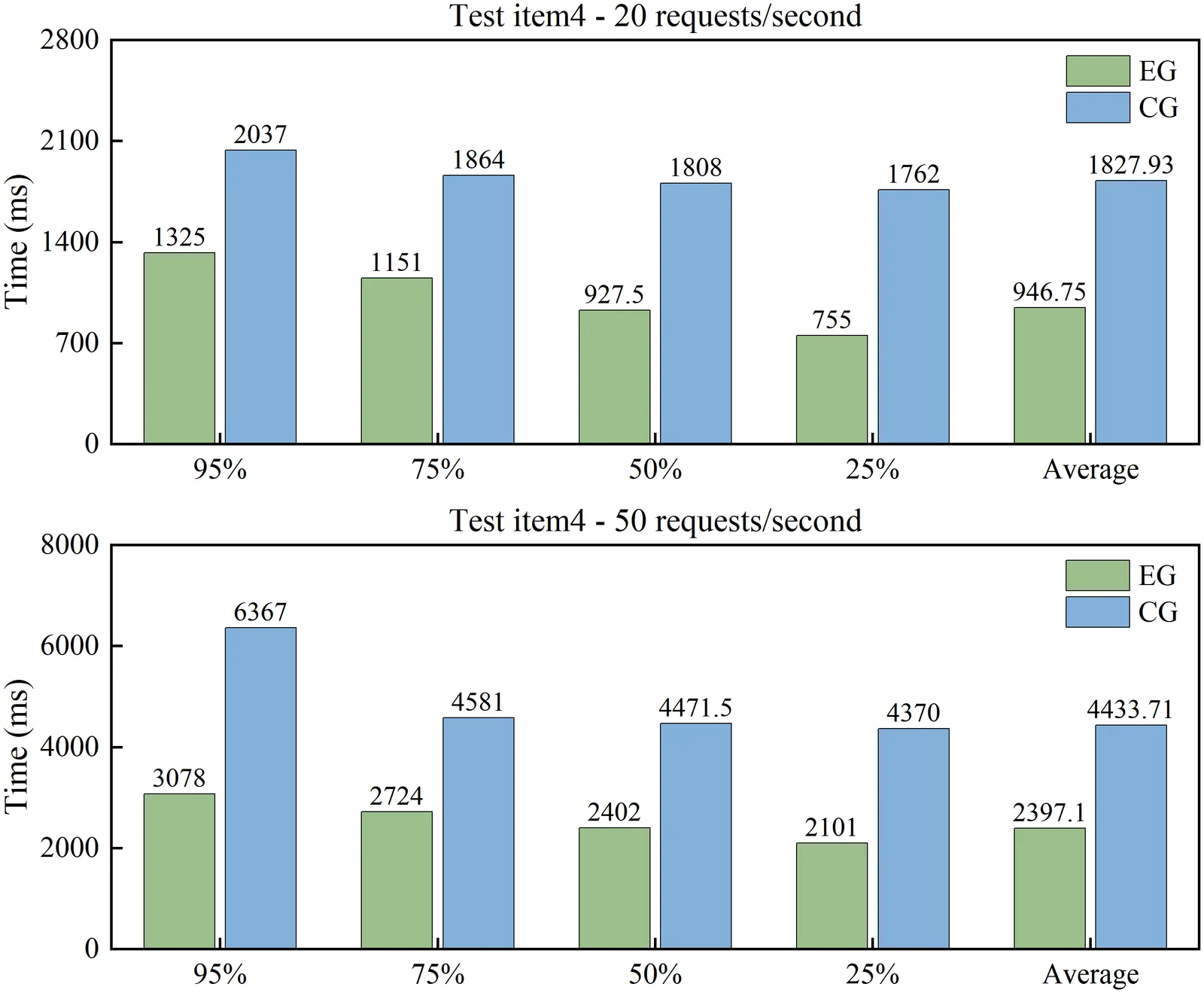
Latency distribution of Test Item 4. Response times are compared between EG and CG under traffic loads of 20 and 50 requests per second.

The performance of Test Item 4 under varying traffic loads is presented in Fig 12. At 20 requests per second, the EG mean latency was 946.75 ms, compared with 1827.93 ms in the CG. At 50 requests per second, the EG mean latency was 2397.10 ms, compared with 4433.71 ms in the CG. Both architectures exhibited stable response times without abrupt spikes.

At 50 requests per second, the CG showed a more pronounced increase at higher percentiles than the EG. This distribution supports the interpretation that the cloud-native deployment handled the higher-load condition more evenly for this workload.

**Fig 13:**
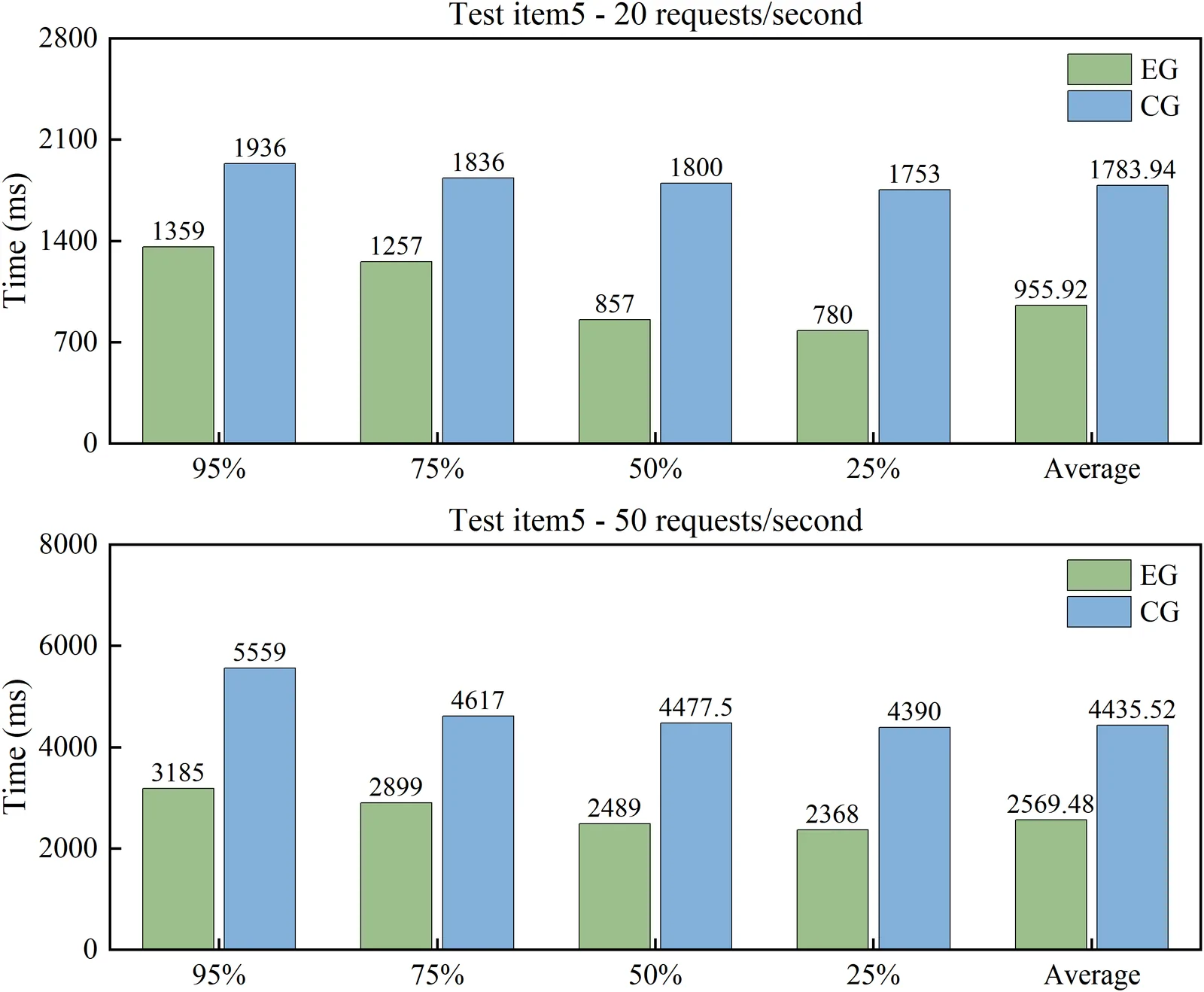
Latency distribution of Test Item 5. Response times are compared between EG and CG under traffic loads of 20 and 50 requests per second.

The performance evaluation of Test Item 5 is illustrated in Fig 13. At 20 requests per second, the EG mean latency was 955.92 ms, compared with 1783.94 ms in the CG. Both architectures showed stable percentile distributions without sudden spikes.

At 50 requests per second, the EG mean latency was 2569.48 ms, compared with 4435.52 ms in the CG. Neither architecture experienced a sudden latency surge, but the CG showed a larger increase at higher percentiles. The result is consistent with the overall observation that the EG maintained lower latency under increased load.

**Fig 14:**
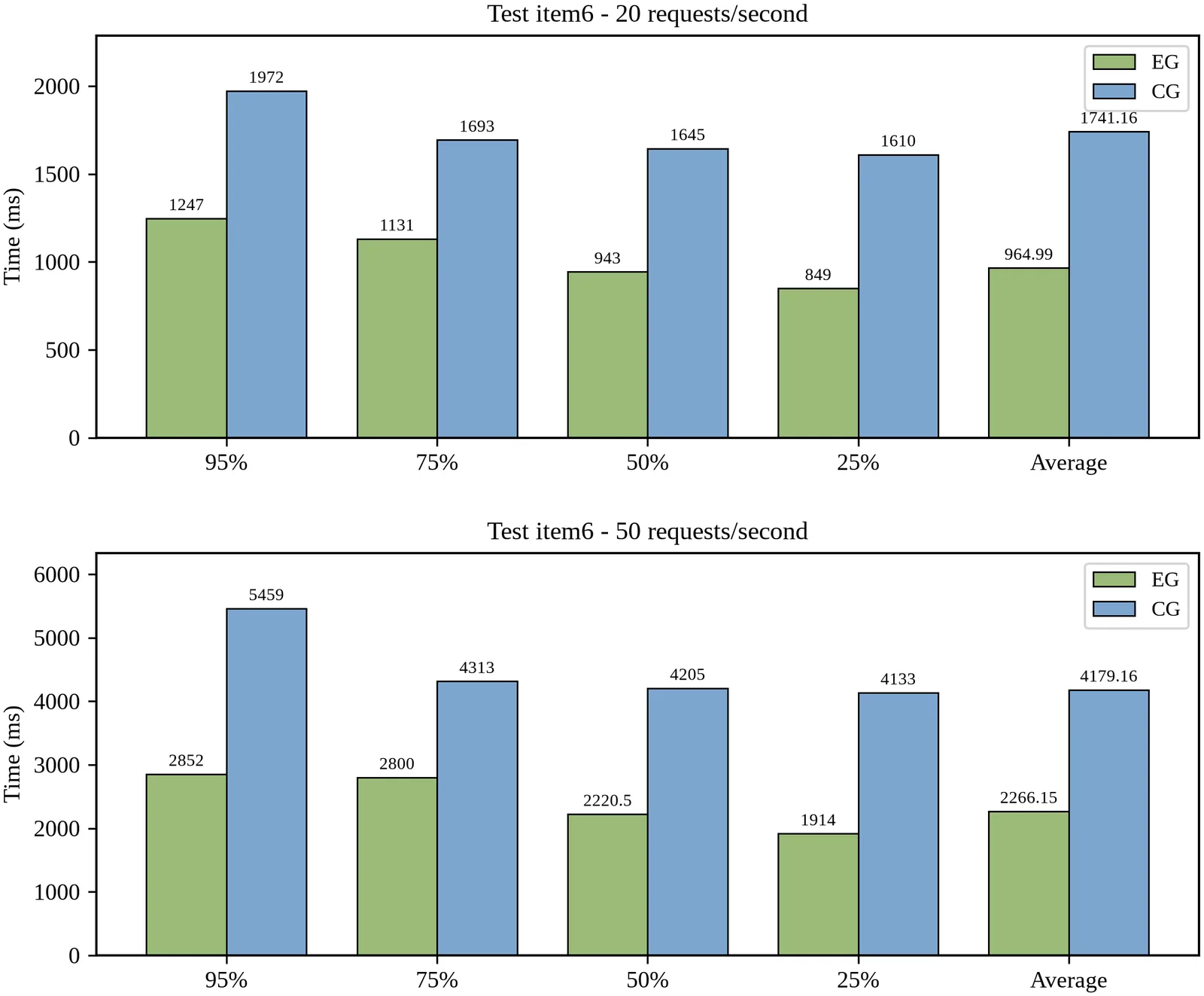
Latency distribution of Test Item 6. Response times are compared between EG and CG under traffic loads of 20 and 50 requests per second.

The performance of Test Item 6 under varying traffic loads is depicted in Fig 14. At 20 requests per second, the EG mean latency was 964.99 ms, compared with 1741.16 ms in the CG. At 50 requests per second, the EG mean latency was 2266.15 ms, compared with 4179.16 ms in the CG. Both architectures remained stable, but the EG maintained lower latency across the reported mean values and most percentile values.

Collectively, the detailed tests show that the mean-latency advantage of the cloud-native deployment appears across all six test items. Tail-latency behavior is more heterogeneous, especially in Test Items 1 and 2. The architectural advantage should therefore be understood as workload- and distribution-dependent rather than as a uniform improvement at every percentile.

## 5 Discussion

As seismic observation networks evolve toward high density, broadband, and dynamic operations, real-time processing of petabyte-scale waveform data has become a bottleneck for earthquake early warning and quick reporting. Under iso-resource constraints, this study found that the Kubernetes-based cloud-native microservice deployment reduced mean response latency relative to a monolithic VM deployment when computing three seismic geometric indices. The mean latency reductions were 47.27% at 20 requests per second and 47.26% at 50 requests per second, corresponding to an approximately 1.90-fold speedup in both load regimes. These gains are interpreted below in relation to virtualization granularity, scheduling path length, isolation strategy, and concurrency handling.

### 5.1 Differences in virtualization granularity and overhead

VMs use hardware-level virtualization, which requires a complete guest operating system and a separate virtualized hardware environment. This deployment pattern can increase runtime overhead and reduce the resources available to the application process. In contrast, the cloud-native architecture is based on operating system-level virtualization, where containers share the host kernel and occupy fewer resources per execution unit. This difference is a plausible explanation for the lower mean latency observed in the EG under the same total hardware configuration.

### 5.2 Efficiency variations in resource scheduling and task response

VMs require scheduling across the host and guest operating systems, whereas cloud-native containers are scheduled directly on the host kernel through the Kubernetes control plane. This shorter execution path may reduce scheduling delay for short-lived waveform quality assessment tasks. In the experiments, the CG showed larger high-percentile latency increases in several cases, especially Test Item 2. Although HPA was not the only factor, the combined effect of container scheduling, replica distribution, and task-lifecycle-aware contraction likely contributed to the observed high-load stability.

### 5.3 Impact of isolation mechanisms on elastic adaptability

The strong isolation of VMs provides instance independence but can make dynamic resource reallocation coarse and costly. The cloud-native architecture adopts process-level isolation with container-level resource limits, allowing finer-grained redistribution of computational tasks across replicas. In the present setting, this design allowed the same total hardware budget to be divided into two independently scheduled algorithm instances. This likely reduced blocking when concurrent requests increased.

### 5.4 Performance advantages under high concurrency

The performance gap between the two architectures was maintained at the higher traffic level. In the VM deployment, all concurrent requests shared a single operating system instance and may have competed for locks, queues, and runtime resources. The cloud-native deployment distributed computational tasks into independently scheduled container replicas, which reduced centralized competition under the tested conditions. This interpretation is consistent with the lower mean latency and the flatter high-percentile behavior observed in several EG test cases.

### 5.5 Scope and limitations

The results should be interpreted within the experimental scope. The evaluation used one 24-hour dataset from 1,000 stations, six test items derived from three geometric indices, two traffic levels, and a fixed 2-core/4 GB resource budget. The experiments measured response latency and latency distributions, but did not directly measure energy consumption, queue length, scheduler internals, or node-level expansion effects. Therefore, the findings support the latency benefit of containerized replica scheduling for the tested seismic quality assessment workloads, but they do not establish universal superiority for all seismic algorithms, deployment scales, or long-duration operational conditions. In addition, the CPU and memory thresholds ($\theta_{cpu}$, $\theta_{mem}$) and the expansion coefficient κ were fixed operational configuration values in this study; a sensitivity analysis of these scaling-strategy parameters was not performed and remains future work. Future work should also test multi-day event sequences, additional quality indicators, larger clusters, and direct resource-utilization traces.

## 6 Conclusion

This study proposed and evaluated a cloud-native computing approach for seismic geometric indices based on Kubernetes orchestration and Docker virtualization. The framework decouples waveform quality assessment into containerized services and combines CPU/memory high-watermark expansion with task-lifecycle-aware contraction. Under equivalent 2-core/4 GB hardware constraints, the cloud-native deployment reduced mean response latency by 47.27% at 20 requests per second and 47.26% at 50 requests per second, corresponding to an approximately 1.90-fold speedup in both settings. These results show that containerized replica scheduling can improve the latency of gap count, gap duration, and availability calculations without changing the underlying quality-metric code. The conclusion is bounded by the tested dataset, workloads, resource budget, and traffic levels. Future research should evaluate larger clusters, longer event sequences, additional quality indicators, and direct resource-utilization traces to determine how the approach generalizes to operational seismic monitoring systems.

## Supporting information

S1 Data Minimal dataset underlying the reported figures and summary statistics. The CSV file contains the 95th, 75th, 50th, and 25th percentile response times and mean response times for all six test items, both concurrency levels, and both experimental groups.
(CSV)
